# A Single-Center Study of the Utility of Bronchoalveolar Lavage in Critically Ill Patients With Haematological Malignancy or Stem Cell Transplants

**DOI:** 10.7759/cureus.50296

**Published:** 2023-12-10

**Authors:** Mohammad Ayaz Khan, Hajar Alhayyan, Hamdan H Aljahdali, Rajkumar Rajendram, Dana Alturaifi, Muhammad Jawad, Sami M Alyami, Hooryia Sher, Ahmed M Almutairi, Majed Alghamdi

**Affiliations:** 1 Department of Medicine, King Abdulaziz Medical City Riyadh, Riyadh, SAU; 2 College of Medicine, King Saud Bin Abdulaziz University for Health Sciences, Riyadh, SAU; 3 Research, King Abdullah International Medical Research Center, Riyadh, SAU; 4 Research and Development, King Abdullah International Medical Research Center, Riyadh, SAU; 5 Department of Biomedical Science, York University, Toronto, CAN

**Keywords:** safety, antimicrobials, bronchoalveolar lavage, stem cells transplant, haematological malignancy

## Abstract

Introduction: The aim of this study was to evaluate the yield of bronchoscopy-guided bronchoalveolar lavage (BAL) and decisions on management of antimicrobials in critically ill patients with hematological malignancy and/or hematological stem cell transplant (HSCT). The safety and tolerance of bronchoscopy were also reported.

Methods: A retrospective cohort study was conducted by reviewing health charts of all adult patients with a hematological malignancy and/or an HSCT who were admitted to the intensive care unit and underwent bronchoscopy and BAL over four years from April 2016 to April 2020 at King Abdulaziz Medical City, Riyadh.

Results: The cohort included 75 critically ill patients. Of these 75 patients, 53 (70.7%) had HSCT (allogenic 66%, autologous 32.1%, haplogenic 3.8%). Computed tomography of the chest was abnormal in all patients. Predominant findings included airspace abnormalities, ground glass opacities, and others. The positive yield was found to be 20% for bacterial, 22% for viral, 21% for fungal, and other organisms were identified in 2%. Although cytology was not performed in 18 patients, malignant cells were identified on BAL in two patients. While the overall mortality of the cohort was high (46.7%), the vast majority (94.7%) tolerated bronchoscopy and BAL without any complications. However, three patients (4%) developed a pneumothorax and one patient bled and developed the acute respiratory distress syndrome post bronchoscopy.

Conclusions: BAL can identify and detect microorganisms directly influencing the clinical care of patients who have received non-invasive diagnostic tests that yielded negative culture results. Bronchoscopy and BAL are generally safe and well tolerated by critically ill patients with hematological malignancy or HSCT.

## Introduction

Patients with haematological malignancy or stem cell transplants are at high risk of developing pulmonary complications [1-3]. These can be life-threatening and are difficult to diagnose. The differential diagnosis of lung infiltrates in this population includes infectious, malignant, and non-infectious causes, such as drug toxicity, graft-versus-host disease, and diffuse alveolar haemorrhage [2]. The optimal diagnostic approach for these patients remains controversial, as conventional methods such as blood cultures, sputum samples, and serological tests have low sensitivity and specificity [3].

Bronchoalveolar lavage (BAL) involves the instillation of sterile saline into a segment of the lung. Aspiration of the BAL sample then allows the collection of alveolar fluid and cells. This fluid can be analyzed for microbiological, cytological, and immunological markers. The BAL procedure may be performed blindly but is usually performed during bronchoscopy via a flexible bronchoscope.

Bronchoalveolar lavage is often used for the diagnosis of pulmonary complications in patients with hematologic malignancies or stem cell transplants. Bronchoalveolar lavage can provide direct evidence of causative agents or pathological processes, but it has important limitations. These include its invasiveness, potential complications, cost, and variable diagnostic yield.

Jorge et al. found that the results of BAL changed management in 57.4% of the cases [1]. Therapeutic interventions included the additions of new antimicrobials to empiric regimens [1]. The findings of Feinstein et al. [2] were similar to those of Jorge et al. [1]. However, other studies reported that survival did not improve despite any change in treatment after a positive result from the analysis of a BAL or transbronchial biopsy [4]. Patients treated for hematological malignancies, in particular those undergoing allogeneic hematopoietic stem cell transplantation (HSCT), are immunosuppressed and have an increased risk of serious infections. Neutropenia, decreased cellular immunity, hypogammaglobulinemia, chemotherapy-induced damage to mucosal barriers, and the frequent use of central venous catheters predispose to such infections [5-6].

There is scarce data about the overall utility of BAL as a diagnostic tool to guide decision-making in this vulnerable cohort and the available data is conflicting. Furthermore, given the trend towards empirical antimicrobial therapy, it is uncertain whether the performance of BAL influences management or changes the outcomes of these patients. Moreover, there are no data on the utility or importance of BAL in patients with hematologic malignancies or stem cell transplantation in Saudi Arabia.

The aim of the study was to evaluate the outcomes and management decisions of patients with hematological malignancies, HSCT or both who developed pulmonary complications and required BAL.

## Materials and methods

Study setting

King Abdulaziz Medical City, Riyadh (KAMCR) is a large regional academic tertiary care center with a total of 1500 beds and a dedicated haemato-oncology service that has a stem cell transplantation program. There are several specialist intensive care units (ICU) with more than 100 beds. When patients with haematological malignancy or stem cell transplantation require organ support, the threshold for admission to a critical care area is low. When indicated, bronchoscopy and BAL may be performed by either an intensivist or a pulmonologist.

Study design

A retrospective cohort study was conducted after approval from the institutional research board (IRB) at King Abdullah International Medical Center, Riyadh, Saudi Arabia (Approval number IRB/2284/22).

Subjects

Patients who underwent bronchoscopy while admitted to an ICU at KAMCR between April 2016 and April 2020 were identified from the records of bronchoscope usage. The electronic healthcare records of these patients were screened to identify those patients with haematological malignancy and/or an HSCT for inclusion in the study. All other patients who underwent bronchoscopy were excluded.

Clinicians who had experience in the field extracted data from the electronic healthcare records of all adult patients with a hematological malignancy and/or an HSCT who were admitted to an ICU at KAMCR and underwent bronchoscopy and BAL between April 2016 and April 2020. 

Data collection

Besides standard demographic data, the authors collected data on smoking status, comorbidities (including infection with the human immunodeficiency virus), type of haematological malignancy, type of HSCT, laboratory blood tests, lung function tests, imaging results, microbial culture results, usage of blood products (e.g., platelet transfusions) and the yield from bronchoalveolar lavage. Diagnoses, changes to treatment, length of stay and mortality were also recorded. These data were used to explore the impact of the BAL on the management and outcomes of the present cohort.

Statistical analyses

Statistical analyses of demographics, laboratory blood results, lung function, imaging, treatments, clinical outcomes, and mortality data have been tabulated. The mean, standard deviation, and range were used to describe continuous data. Frequencies and percentages were used to describe categorical data. All statistical analyses were performed using Excel version 2016 (Microsoft, Redmond, WA, USA).

## Results

Table 1 presents the demographic data of the patients (N=75; male 48/75, 64%; mean age 40 years) who underwent bronchoscopy at King Abdulaziz Medical City, Riyadh between February 2016 and April 2020. The vast majority (92%; 69/75) were non-smokers. Nearly one-third of the cohort (29.3%; 22/75) had not had HSCT. The ratio of allogenic to autologous transplants was 66.0% (35/53) to 32.1% (17/53), while 3.8% (2/53) were haplogenic. Only one patient was HIV positive but the majority (98.7%; 74/75) of the cohort was on immunosuppressants.

**Table 1:** Demographic data (N = 75) Continuous data are presented as mean and range while categorical data are presented as frequency and percentage. HIV: human immunodeficiency virus; HSCT: hematological stem cell transplant.

Characteristic	Number (%)
Age (years)	
Mean (range)	40 (16-79)
SD	18.0
Gender	
Male	48 (64.0%)
Female	27 (36.0%)
Smoking	
Non-smoker	69 (92.0%)
Current	4 (5.3%)
Ex-smoker	2 (2.7%)
HIV	
Negative	74 (98.7 %)
Positive	1 (1.3%)
HSCT	
Yes	53 (70.7%)
No	22 (29.3%)
Type of Transplant	
Autologous	16 (21.3%)
Allogenic	35 (46.7%)
Haplogenic	2 (2.7%)
None	22 (29.3%)
Immunosuppression	
Yes	74 (98.7 %)
No	1 (1.3%)

Table 2 lists the patients’ hematological diagnosis and co-morbidities (N=75). The four most common diagnoses were acute myeloid leukaemia (AML; 30.7%), diffuse large B-cell lymphoma (DLBCL; 10.7%), Hodgkin's lymphoma (HL; 9.3%), and multiple myeloma (MM; 8.0%). Within this group, 61.3% of patients (46/75) had no co-morbidities while 16% of patients (12/75) had more than two co-morbidities. The three most common co-morbidities were diabetes mellitus (DM; 14.7%, N=11), hypothyroidism (6.7%, N=5), and hypertension (HTN; 5.3%, N=4).

**Table 2:** The hematological disease diagnoses and co-morbidities HIV: human immunodeficiency virus; CKD: chronic kidney disease; DM: diabetes mellitus; HTN: hypertension; IHD: ischaemic heart disease; HF: heart failure; BMF: bone marrow failure.

Characteristic	Number
Hematological Disease Diagnosis	
Hodgkin’s Lymphoma	7 (9.3%)
Multiple Myeloma	6 (8.0%)
Diffuse Large B-cell Lymphoma	8 (10.7%)
Acute Myeloid Leukemia	23 (30.7%)
Pre-B-cell Acute Lymphocytic Leukemia	4 (5.3%)
Acute lymphocytic Leukemia	2 (2.7%)
T-cell acute Lymphocytic Leukemia	5 (6.7%)
Sickle cell Anaemia Post Stem Cell Transplant	5 (6.7%)
B-cell Acute Lymphocytic Leukemia	4 (5.3%)
Polymyositis	3 (4.0%)
Plasma Cell Leukemia	2 (2.7%)
Burkitt Lymphoma	2 (2.7%)
Congenital BMF/Aplastic Anemia with Short Telomere Syndrome	1 (1.3%)
Sickle Cell Anemia Pre-Stem Cell Transplant	1 (1.3%)
Non-Hodgkin Lymphoma	2 (2.7%)
Co-morbidities	
None	46 (61.3%)
Asthma	1 (1.3%)
Bronchiectasis	1 (1.3%)
Chronic Graft Versus Host Disease	1 (1.3%)
Chronic Kidney Disease (CKD)	1 (1.3%)
Diabetes Mellitus (DM)	6 (8.0%)
DM, Hyperlipidaemia	1 (1.3%)
DM, Hypertension (HTN), Ischaemic Heart Disease (IHD), Heart Failure (HF)	1 (1.3%)
DM, Inferior Vena Cava Thrombosis	2 (2.7%)
DM, Rheumatoid Arthritis	1 (1.3%)
Fanconi Anemia	1 (1.3%)
Glucose 6 Phosphate Dehydrogenase Deficiency	1 (1.3%)
HIV, RHD, HF, Epilepsy	1 (1.3%)
HTN, IHD, Hypothyroidism	1 (1.3%)
HTN, Atrial Fibrillation	1 (1.3%)
HTN, CKD	1 (1.3%)
Hypothyroidism	3 (4.0%)
Hypothyroidism, Cryptogenic Organising Pneumonia	1 (1.3%)
Myelofibrosis	1 (1.3%)
Obesity	1 (1.3%)
Pulmonary Embolism, Hepatitis B	1 (1.3%)
Seizure Disorder, Vitiligo	1 (1.3%)

Table 3 shows lung function data and the results of laboratory blood tests. Lung function tests were not performed in 27 patients. In the remaining 48 patients (64%) who had lung function tests, the mean forced expiratory volume over 1 second (FEV1) was 77.4% of predicted, the mean forced vital capacity (FVC) was 76.6% of predicted (mean FEV1/FVC ratio was 95.1%). The mean total lung capacity (TLC) was 74.6% of the predicted. The mean C-reactive protein (CRP) was 144 mg/dl, and the mean white cell count (WCC) was 11.4 × 109/L (neutropenia 27/75, 36%). Platelet transfusions were required for 44% of patients (33/75). Abnormalities were identified on the computed tomography (CT) chest scan in all patients. The predominant CT patterns observed were airspace abnormality (consolidation, airspace abnormality, airspace opacities, and air bronchogram), ground glass abnormality (ground glass and ground-glass opacities (GGO)), and others (tree-in-bud opacities, halo signs, micronodulation, nodular abnormality, and crazy paving). Nearly half of the patients (48%; 36/75) had more than two different patterns of abnormalities.

**Table 3:** Laboratory blood tests and lung function FEV1: forced expiratory volume over one second; FVC: forced vital capacity; TLC: total lung capacity.

Characteristic	Number
FEV1	
Mean (Range)	77.4% (27-121)
SD	21.0
FVC	
Mean (Range)	76.6% (34-127)
SD	18.7
FEV1/FVC Ratio	
Mean (Range)	95.1% (48.94-115)
SD	14.1
TLC	
Mean (Range)	74.7% (48-105)
SD	13.3
C Reactive Protein	
Mean (Range)	144.1 (3-509) mg/dl
SD	145.6
White Cell Count	
Mean (Range)	11.4 (0.02-235) × 10^9^/L
SD	28.2
Neutropenic	
Yes	27 (36%)
No	48 (64%)
Platelets	
Mean (Range)	113.1 (4-410) × 10^9^/L
SD	103.0
Platelet Transfusion	
Yes	33 (44%)
No	42 (56%)
Units of Platelets Tranfused	
Mean (Range)	4.48 (0-18)
SD	5.6
Findings on Computed Tomography of the Chest	
Airspace Abnormality	7 (9.3%)
Airspace Abnormality and Others	7 (9.3%)
Airspace Abnormality and Ground Glass Abnormality	16 (21.3%)
Ground Glass Abnormality	9 (12.0%)
Ground Glass Abnormality and Others	13 (17.3%)
Others	10 (13.3%)
Miscellaneous Group	13 (17.3%)

Table 4 shows the results of the cohort’s non-invasive microbial investigations. Blood cultures were negative in 89.3% (67/75). However, blood cultures were not taken from three patients. The organisms isolated from blood cultures in the remaining patients were *Pseudomonas* *aeruginosa* (two patients; 2.7%), *Klebsiella* *pneumoniae* (one patient; 1.3%), methicillin-resistant *Staphylococcus aureus* (MRSA; one patient; 1.3%), and extended-spectrum beta-lactamase (ESBL) *Escherichia coli *(one patient; 1.3%). Sputum cultures were negative in 64% (48/75), but were not collected from 18 patients (24%). The pathogens isolated from the sputum cultures were *Pseudomonas* (one patient; 1.3%), *Mycobacterium tuberculosis* (MTB; one patient; 1.3%), respiratory syncytial virus (one patient; 1.3%), *Stenotrophomonas maltophilia* (one patient; 1.3%), *Candida tropicalis* (one patient; 1.3%), *Klebsiella pneumoniae* (one patient; 1.3%), *Aspergillus fumigatus* (one patient; 1.3%), yeast (one patient; 1.3%), and MRSA (one patient; 1.3%). Furthermore, 52% of the viral multiplex polymerase chain reaction (PCR) tests performed on nasopharyngeal swabs were negative, but nasopharyngeal swabs were not collected from 21 patients (28%). The most common viruses detected from nasopharyngeal swabs were human rhinovirus/enterovirus (six patients). The serum galactomannan index was negative in 74.7% of the cohort (56/75).

**Table 4:** Results of non-invasive investigations COVID-19: coronavirus disease 2019; ESBL: extended-spectrum beta-lactamase; MRSA: methicillin-resistant *Staphylococcus aureus*; RSV: respiratory syncytial virus.

Characteristic	Number
Blood Culture Results	
Negative	67 (89.3%)
Not Done	3 (4.0%)
Pseudomonas aeruginosa	2 (2.7 %)
Klebsiella pneumonia	1 (1.3%)
MRSA	1 (1.3%)
ESBL *Escherichia coli*	1 (1.3%)
Sputum Culture Results	
Negative	48 (64.0%)
Not Done	18 (24.0%)
Pseudomonas aeruginosa	1 (1.3%)
Mycobacterium tuberculosis	1 (1.3%)
RSV	1 (1.3%)
Stenotrophomonas maltophilia	1 (1.3%)
Candida tropicalis	1 (1.3%)
Klebsiella pneumoniae	1 (1.3%)
Aspergillus fumigatus	1 (1.3%)
Yeast	1 (1.3%)
MRSA	1 (1.3%)

Characteristic	Number
Nasopharyngeal Swabs	
Negative	39 (52.0%)
Not Done	21 (28.0 %)
Human Rhinovirus/Enterovirus	6 (8.0%)
Human Rhinovirus	2 (2.7%)
RSV and Rhinovirus	1 (1.3%)
RSV	1 (1.3%)
Rhinovirus and Parainfluenza	1 (1.3%)
Coronavirus NL63	1 (1.3%)
Human Metapneumovirus	1 (1.3%)
COVID-19 Negative, Others Not Done	1 (1.3%)
Adenovirus	1 (1.3%)
Serum Galactomannan Index	
Negative	56 (74.7%)
Positive	5 (6.7%)
Not Done	14 (18.7%)

Table 5 shows the results of the microbiological investigations performed on the BAL. As shown in Table 5, the three main groups of pathogens identified in the BAL were bacteria (14/75, 18.7%), viruses (16/75, 35.6%), and fungi (16/75, 35.6%). The most common bacteria were *Klebsiella pneumoniae* (4/75; 5.3%) and *Pseudomonas aeruginosa* (3/75; 4.0%). The most common fungi were *Candida glabrata* (5/75; 6.7%), *Candida dubliniensis* (3/75; 4.0%) and *Candida albicans* (3/75; 4.0%).

**Table 5:** Results of the microbiological investigations performed on the bronchoalveolar lavage (BAL)

Characteristic	Number
BAL Bacterial Culture Result	
Negative	60 (80.0%)
Not Done	1 (1.3%)
Candida	1 (1.3%)
Nocardia	1 (1.3%)
Streptococcus pneumoniae	1 (1.3%)
Pseudomonas aeruginosa	3 (4.0%)
Serratia marcescens	1 (1.3%)
Enterococcus faecium	1 (1.3%)
Stenotrophomonas maltophilia	1 (1.3%)
Klebsiella pneumoniae	4 (5.3%)
Haemophilus influenzae	1 (1.3%)
BAL Viral Multiplex Polymerase Chain Reaction (PCR) Result	
Negative	42 (56.0%)
Not Done	17 (22.7%)
Respiratory Syncytial Virus	4 (5.3%)
Human Rhinovirus	2 (2.7%)
Influenza A	2 (2.7%)
RSV	1 (1.3%)
Coronavirus	2 (2.7%)
Parainfluenza Virus	1 (1.3%)
Human Rhinovirus/Enterovirus	2 (2.7%)
Human Metapneumovirus	1 (1.3%)
Adenovirus	1 (1.3%)
BAL Fungal Culture Result	
Negative	58 (77.3%)
Not Done	1 (1.3%)
Candida glabrata	5 (6.7%)
Candida dubliniensis	3 (4.0%)
Candida albicans	3 (4.0%)
Candida troplicalis	1 (1.3%)
Candida krusei	2 (2.7%)
Aspergillus niger	1 (1.3%)
Saccharomyces cerevisiae	1 (1.3%)

Table 6 shows the cytological analyses performed on BAL. This tested for other organisms, cell counts and white cell differential, CD4/CD8 T cells, and malignant cells. Other organisms detected from the BAL were human rhinovirus (1/75, 1.3%) and *Asperigillus* (1/75, 1.3%). Cell counts revealed that 41.3% (31/75) of the BALs were neutrophilic, 38.7% (29/75) were monocytic, and 20% (15/75) were lymphocytic. Malignant cells were only detected in the BAL from two patients (2.7%), but cytological assessment for malignancy was not performed in a quarter of the patients (18/75; 24%).

**Table 6:** Cytological analyses performed on the bronchoalveolar lavage (BAL).

BAL Other Organisms	Number (%)
None	73 (97.3%)
Human Rhinovirus	1 (1.3%)
Asperigillus	1 (1.3%)
BAL Cell Type	
Lymphocytic	15 (20.0%)
Neutrophilic	31 (41.3%)
Monocytic	29 (38.7%)
CD4/CD8 T Cells	
Negative	2 (2.7%)
Positive	1 (1.3%)
Not Done	67 (89.3%)
Low	4 (5.3%)
Normal	1 (1.3%)
BAL Malignant Cells	
Negative	55 (73.3%)
Positive	2 (2.7%)
Not Done	18 (24.0%)

Table 7 shows the patients’ diagnosis, treatment, outcomes, and bronchoscopy-related complications. The present cohort received six types of treatment: antibiotic, antifungal, antiviral, anti-*Pneumocystis carinii* pneumonia (PCP), anti-TB, and steroids. After bronchoscopy and BAL, antibiotics were initiated in 22.7% of patients (17/75), antibiotics were changed in 32% (24/75), and in 34.7% (26/75) antibiotics were stopped. Antifungal treatment was started in 28% (21/75) and stopped in 14.7% (11/75). Treatment for PCP was initiated in 10.7% of patients (8/75) and one patient (1.3%) received anti-TB treatment. Steroids were started in one patient (1.3%). The most common final diagnoses were pneumonia (11/75, 14.7%) and presumed fungal sepsis (11/75, 14.7%). There were very few bronchoscopy-related complications (4/75; 5.3%) and 53.3% of patients (40/75) survived to hospital discharge. 

**Table 7:** Treatment, diagnosis, and outcome of the cohort ARDS: acute respiratory distress syndrome; GVHD: graft versus host disease; PCP: Pneumocystis carinii pneumonia.

Treatment, Diagnosis, and Outcomes	Number (%)
Antibiotic Withdrawal	26 (34.7%)
Antifungal Withdrawal	11 (14.7%)
Antibiotic Started	17 (22.7%)
Antifungal Started	21 (28.0%)
Antibiotic Change (Narrow Spectrum Agent)	24 (32.0%)
Antiviral Started	5 (6.7%)
Treatment for PCP Started	8 (10.7%)
Anti Tuberculous Therapy Started	1 (1.3%)
Other Treatment	
None	74 (98.7%)
Steroids	1 (1.3%)
Final Diagnosis	
Bacterial Sepsis Unknown Origin	3 (4.0%)
Bacterial Sepsis	1 (1.3%)
Bronchoscopy for Pulmonary Hemorrhage, Scope Negative. Treated as TRALI and HAP	1 (1.3%)
Fungal Pneumonia	8 (10.7%)
Fungal Sepsis	9 (12.0%)
Fungal Sepsis Presumed	11 (14.7%)
GVHD	3 (4.0%)
Invasive Aspiriligillosis	1 (1.3%)
Invasive Fungal Infection	1 (1.3%)
Mixed Sepsis Infection	1 (1.3%)
Nocardia	1 (1.3%)
Organizing Pneumonia	1 (1.3%)
PCP Presumed	1 (1.3%)
Pneumonia	11 (14.7%)
Streptococcal Pneumonia	1 (1.3%)
Tuberculosis	1 (1.3%)
Viral Pneumonia	2 (2.7%)
Viral Sepsis	1 (1.3%)
Patient Outcome	
Alive	40 (53.3%)
Died	35 (46.7%)
Bronchoscopy Complications	
None	71 (94.7%)
Pneumothorax	3 (4.0%)
ARDS, Bleeding	1 (1.3%)

## Discussion

The present retrospective study determined that the diagnostic yield of BAL for patients with respiratory diseases was around 60%. This allowed the identification of bacterial, viral, and fungal infections. Pulmonary infiltrates are common in unwell patients with hematological malignancy. This finding is associated with high rates of morbidity and mortality [7-11]. The differential diagnosis for the etiology of these infiltrates is broad [12]. However, around 70% are infective in nature.

BAL is a well-established method of identifying the cause of pulmonary infiltrates. The routine use of prophylactic antibacterial agents, antiviral agents, and antifungal agents in combination with empirical antimicrobial therapy, before bronchoscopy, may reduce the diagnostic yield [12,13]. These observations are similar to the findings in previous studies, showing a diagnostic yield of 31% to 83% depending on the underlying disease [14,15]. The BAL cultures were positive for viral and fungal infections in 32 cases (42%). Bacterial cultures were positive in 14 cases (18.6%). Those findings are similar to the study by Ghandili et al. [16].

In contrast, non-invasive diagnostic methods identified only 29 positive microbiological results. These were mainly due to respiratory viral swabs (15 cases) or sputum cultures (nine cases). However, the respiratory viral swabs were not done in 21 cases. Serum galactomannan index was positive in only five cases (6.7%) but was not checked in 14 cases (18.7%) before the BAL. Only one sputum sample was positive for Mycobacteria.

In nearly 80% of cases, the BAL was negative for bacteria, which can explain why empirical antibiotics were not effective in those patients. However, the bacterial cultures may be falsely negative in the setting of ongoing treatment with empirical or pre-emptive broad-spectrum antibiotics that were administered to the majority of the cases in the present cohort.

In almost half of the cases, negative BAL results resulted in the withdrawal of antibiotics and antifungal agents. The positive BAL results also led to a modification in therapeutic regimes in 51 cases (68%), including the addition of antifungal or antiviral agents and the change of antibiotics to one with a narrower spectrum (32%). The negative BAL analysis allowed discontinuation of unnecessary therapies, minimizing the adverse effects and possible development of multi-drug resistant organisms. The findings are consistent with those of previous studies which have reported that bronchoscopy-guided BAL can inform complex antimicrobial stewardship decisions leading to the adjustments of therapies in patients with hematological disease [5,6,17-19]. However, the rates of therapeutic interventions reported in previous studies have varied widely, ranging from 5% to 50% [5,6,17-19].

In the present study, malignant cells were identified in the BAL of only two patients. These findings were related to the primary hematological diagnosis. This is similar to previous studies that confirmed pulmonary infections as the primary cause for the pulmonary infiltrates in this cohort of patients [20-22].

In the present study, several BAL analyses were non-diagnostic. Therefore, regular re-evaluation of the usefulness and cost-effectiveness of such interventions is important. Bronchoscopy and BAL seemed to be safe in the present cohort. Only three patients had pneumothorax and one developed acute respiratory distress syndrome (ARDS) and bleeding post bronchoscopy. Nearly half of the patients died from their primary pathologies. No deaths were directly attributed to the performance of the bronchoscopic procedure itself.

Limitations

The main limitations of the present study are its retrospective design with the potential for missing data as well as selection bias. The collection of data from a single institution and the relatively small sample size also have the potential for overestimation of the effect. Patient selection may have been affected by the lack of precisely defined criteria for the performance of BAL in critically ill patients with hematological malignancy and or HSCT. The positive yield of BAL might have been closer to the non-invasive diagnostic methods e.g., respiratory viral multiplex PCR on nasopharyngeal swab, had that been performed in all of the cases observed. The lack of a controlled group is also a potential limitation of our study.

## Conclusions

Bronchoscopy and BAL are generally safe and well tolerated by critically ill patients with hematological malignancy or HSCT. The bronchoalveolar lavage can detect and identify organisms. It can have a direct impact on the clinical management of patients with negative cultures from non-invasive diagnostic tests. However, the mortality of critically ill patients with hematological malignancy and/or HSCT remains high. Multicenter randomized controlled trials are required to determine the actual impact of BAL on the management of this complex group of patients.
